# Expression of dementia biomarkers in Appalachian and non-Appalachian ELVO patients during thrombectomy

**DOI:** 10.3389/fnins.2025.1672803

**Published:** 2025-11-11

**Authors:** Neha Anil, Christopher J. McLouth, Hunter S. Hazelwood, Elise Dahlke, Jacqueline A. Frank, Nathan Millson, Mais Al-Kawaz, Jordan P. Harp, Will Cranford, Shivani Pahwa, David Dornbos, Justin F. Fraser, Keith R. Pennypacker

**Affiliations:** 1 College of Medicine, University of Kentucky, Lexington, KY, United States; 2 Department of Neurology, University of Kentucky, Lexington, KY, United States; 3 Department of Biostatistics, University of Kentucky, Lexington, KY, United States; 4 Center for Advanced Translational Stroke Science, University of Kentucky, Lexington, KY, United States; 5 Department of Radiology, University of Kentucky, Lexington, KY, United States; 6 Department of Neurosurgery, University of Kentucky, Lexington, KY, United States; 7 Department of Neuroscience, University of Kentucky, Lexington, KY, United States

**Keywords:** Appalachia, stroke, VCID, dementia, post stroke cognitive impairment, biomarkers

## Abstract

**Background/Context:**

Vascular Cognitive Impairment and Dementia (VCID) affects 25-30% of stroke patients and includes cognitive impairments caused by vascular injury, such as post-stroke dementia. Rehabilitation has the potential to improve the quality of life for patients at risk of developing dementia. However, there is currently no reliable method to identify those at risk of dementia after a stroke. Several biomarkers, including ADRD (Alzheimer’s disease and related dementias) biomarkers (Ab, tau, NfL, and GFAP) and angiogenic factors (VEGF, Flt-1, Tie-2, PIGF, and FGF) have been associated with the development of dementia.

Populations in Appalachia experience a higher incidence of stroke and related mortality compared to other groups. Given the elevated stroke rates in Appalachian communities, this study aims to investigate potential proteomic differences between patients from Appalachian and non-Appalachian counties. The primary goal of the study is to characterize the expression of post-stroke cognitive dementia biomarkers and to explore differences in the proteomic profiles of Appalachian and non-Appalachian populations.

**Methods/Approach:**

Sample Collection: The Blood and Clot Thrombectomy Registry Collaborative (BACTRAC) protocol, established by Fraser and colleagues, introduces a novel method for analyzing stroke by collecting intracranial blood samples from patients undergoing mechanical thrombectomy. During the procedure the thrombus and blood samples from areas distal and proximal to the thrombus are collected and undergo proteomic analysis (Fraser et al.). Additional demographic and clinical information are collected from electronic health records. The control data was obtained from arterial blood collected during diagnostic angiograms from patients with cerebrovascular disease.

**Data Analysis:**

Propensity score models were used to perform a one-to-one match between stroke and control patients on age, sex, BMI, hypertension, and hyperlipidemia resulting in groups that were balanced on these measured prognostic characteristics. A Wilcoxon rank sum test was then used to assess differences in the 12 ADRD biomarkers.

**Results:**

Compared to the controls, stroke patients had significantly higher levels of GFAP. The control patients had significantly higher levels of AB40, AB42, and VEGFA. In the Appalachian patient population, the control patients also had significantly higher levels of AB40, AB42, and VEGFA. Additionally, the Appalachian stroke patients had higher GFAP. In the non-Appalachian population only GFAP was significantly different between stroke and control groups, with it being elevated in the stroke group.

**Conclusion:**

There was a notable difference in the levels of certain ADRD biomarkers between stroke patients and control patients. Specifically, in Appalachian populations, stroke patients showed significant differences in multiple ADRD biomarkers (AB40, AB42, and GFAP) compared to controls, a pattern not seen in non-Appalachian stroke patients, where only GFAP levels increased. This difference in ADRD biomarkers observed in Appalachian stroke patients could be attributed to a combination of socioeconomic and environmental factors unique to the Appalachian region.

## 1 Introduction

Ischemic stroke is a leading cause of mortality in the United States with the highest burden of disease concentrated in the southeastern region of the country known as the “Stroke Belt” (Tsao et al., 2022). As of 2018, the stroke belt had a 22% higher rate of stroke mortality compared to non-stroke belt regions (Parcha et al., 2021) Kentucky is one of multiple states that make up the stroke belt region (Howard and Howard, 2020). Some of the highest incidences of stroke occur in several Eastern Kentucky Appalachian counties (K.D.F.P. Health, 2024), representing some of the most economically depressed counties in the state. These Eastern Kentucky counties have low access to healthcare and high rates of stroke comorbidities such as hypertension, diabetes, and obesity (Kitzman et al., 2019).

Stroke is also a leading cause of disability in the United States (Stroke Facts, 2024) with 25–30% of stroke patients developing vascular cognitive impairment and dementia (VCID), a broad category of cognitive impairments caused by vascular injury including post-stroke dementia (Kalaria et al., 2016). A previous study, using a data registry containing proteomic data gathered from stroke patients at the time of mechanical thrombectomy (MT), found that Appalachian patients undergoing MT for emergent large vessel occlusions strokes (ELVO) have worse cognitive outcomes compared to non-Appalachian patients (McLouth et al., 2024).

Research suggests that ischemic stroke leads to dementia via chronic upregulation of pro-inflammatory cascades, dysregulation of angiogenic factors, and enhancement of Alzheimer’s disease (AD) pathology (Iadecola and Anrather, 2025). Ischemic injury causes increased permeability of the blood brain barrier (BBB) and upregulates acute inflammatory proteins such as glial fibrillary acidic protein (GFAP). GFAP is a nonspecific identifier of vascular injury and increased plasma levels of GFAP have been found among patients with AD (Benedet et al., 2021; Verberk et al., 2020). Further contributions to a pro-inflammatory environment may be caused by angiogenic factors such as fibroblast growth factor (FGF), vascular endothelial growth factor (VEGF), placental growth factor (PIGF), and vascular endothelial growth factor receptor 1 (Flt-1) which have been implicated in the pathogenesis of VCID (Linton et al., 2021; Winfree et al., 2025; Hinman et al., 2023). Although generally regarded as neuroprotective (Fang et al., 2023), some evidence suggests that post-stroke angiogenesis can result in leaky vasculature and create pathways for additional inflammatory mediators to reach the infarcted tissue (Rust, 2020; Yang and Torbey, 2020; Kanazawa et al., 2019; Zhang et al., 2000). Another post-stroke change involves upregulation of Alzheimer’s disease and related dementia (ADRD) biomarkers including amyloid beta (Aβ), tubulin associated unit (tau), and neurofilament light chain (NfL; Gustaw-Rothenberg et al., 2010). These biomarkers hold potential to be used as predictive markers of VCID after stroke.

Given the high incidence of stroke and worsened cognitive outcomes of patients from Eastern Appalachian Kentucky counties, there is a need to identify biomarkers predictive of post-stroke dementia, to improve patient care and decrease disease burden. Previous study analyzed arterial blood samples from Appalachian and non Appalachia patients to look for differences in inflammatory protein expression and associated functional outcomes between the two populations. Out of 184 proteins analyzed, 11 were identified as being differentially associated with post-stroke outcomes based on Appalachian status of the patients. Of these, 2 proteins, leukemia inhibitory factor receptor (LIFR) and angiogenin (ANG), were negatively correlated with the Montreal Cognitive Assessment (MoCA) score at discharge in non-Appalachian patients (McLouth et al., 2024). To expand upon these previous findings, this study has 2 aims. The first is to characterize the early time-point expression of VCID and ADRD biomarkers between stroke and cerebrovascular disease (CVD) controls. The second aim is to compare the biomarker expression between Appalachian and non-Appalachian populations overall and within stroke and CVD control patients.

## 2 Methods

### 2.1 Sample collection

The Blood and Clot Thrombectomy Registry and Collaboration (BACTRAC) is a continuously enrolling tissue bank that contains proteomic and demographic data from patients undergoing cerebrovascular procedures such as mechanical thrombectomy (MT) and angiography. Demographic data in the registry includes sex, age, county, and comorbidities. Patients who were pregnant, incarcerated, had pre-existing inflammatory cerebrovascular disease, or unable to provide informed consent were excluded from the study. Additional information on BACTRAC and its methodology has been previously published (Fraser et al., 2019).

During MT, samples of thrombus, blood distal to the thrombus, and blood proximal to the thrombus are collected from the patient and processed in a laboratory space adjacent to the Neurointerventional Radiology (NIR) angiography suite per the BACTRAC protocol (Fraser et al., 2019). The blood samples were divided into 500 ul aliquots and inverted 10 times to ensure thorough mixing and prevent clotting. The samples were then centrifuged at 2000 g for 15 min in a 22R centrifuge (Beckman Coulter Inc.; Indianapolis Indiana). After centrifugation 200 ul of plasma was extracted from the sample and placed in separate cryogenic storage tubes which were then stored at −80° C until analysis. The analysis was done using several methods for each protein: Meso Scale Discovery V PLEX Angiogenesis Panel 1 (human), Quanterix (Simoa Technology) Neurology 3-Plex A Advantage Kit (AB40, AB42, Tau), Neurology 2-Plex B (GFAP, NF-l) Assay kit, pTau-181 Advantage V2.1 kit. Intra- and inter-assay coefficients of variation (CV) were not assessed for this analysis. The resulting proteomic data was then input into the secure BACTRAC REDCap Database (Gustaw-Rothenberg et al., 2010). Although blood samples proximal and distal to the blood clot were collected, this study focuses on data from the blood proximal to the thrombus. The quantity of blood collected distal to the thrombus was not adequate for analysis.

Control samples were obtained from individuals undergoing diagnostic angiograms for CVD such as arteriovenous malformations (AVM), arteriovenous fistulas, carotid stenosis, or aneurysms. The same exclusion criteria used for the stroke patients were used in the control patient population. Arterial blood was processed using identical methodology as described for the stroke samples. Biomarkers analyzed are listed in Table 1.

**Table 1:** Biomarkers and rationale for selection.

Biomarkers	Effect
A β 40 and A β 42	Aggregates result in neurodegeneration in AD and levels are elevated in patients with cognitive impairment (Chen et al., 2017; Olsson et al., 2016).
Tau and pTau181	Elevated tau has also been implicated in cognitive dysfunction following stroke and AD (Olsson et al., 2016; Grande et al., 2025).
NfLight	Elevated NfL is linked to cognitive decline and neurodegeneration (Olsson et al., 2016; Grande et al., 2025)
GFAP	Biomarker of neuroinflammation that is upregulated AD patients (Grande et al., 2025)
bFGF	Increased levels associated with cognitive impairment and AD (Zhao et al., 2025).
FlT1	A VEGF receptor that has been elevated in AD patients (Winfree et al., 2025).
PlGF	Elevation associated with cognitive impairment (Hinman et al., 2023; Yang et al., 2024).
VEGF-A	Decreased plasma levels associated with dementia (Yang et al., 2024)

### 2.2 Statistical analysis

Propensity scores were employed to match stroke and control patients using the following predictors in the propensity model: age, sex, body mass index, hypertension, and hyperlipidemia. An exact match was performed on sex, and the propensity score was used to match stroke and control patients using a conservative caliper size of 0.2. Following the propensity score match, between-group differences in the 12 ADRD biomarkers were assessed using a Wilcoxon rank sum test. Due to the biomarkers’ non-normal distribution Cliff’s delta, a non-parametric effect size, was used to quantify the magnitude of between group differences, and is a common language effect size interpreted as the probability that a randomly sampled observation from one group is larger than a randomly sampled observation from the other (Cliff, 1993) All statistical analyses were performed using SAS v 9.4 (SAS Institute, Inc., Cary, NC). A *p*-value ≤ 0.05 was used for statistical significance.

## 3 Results

### 3.1 Matched patient demographic data

**Figure 1:**
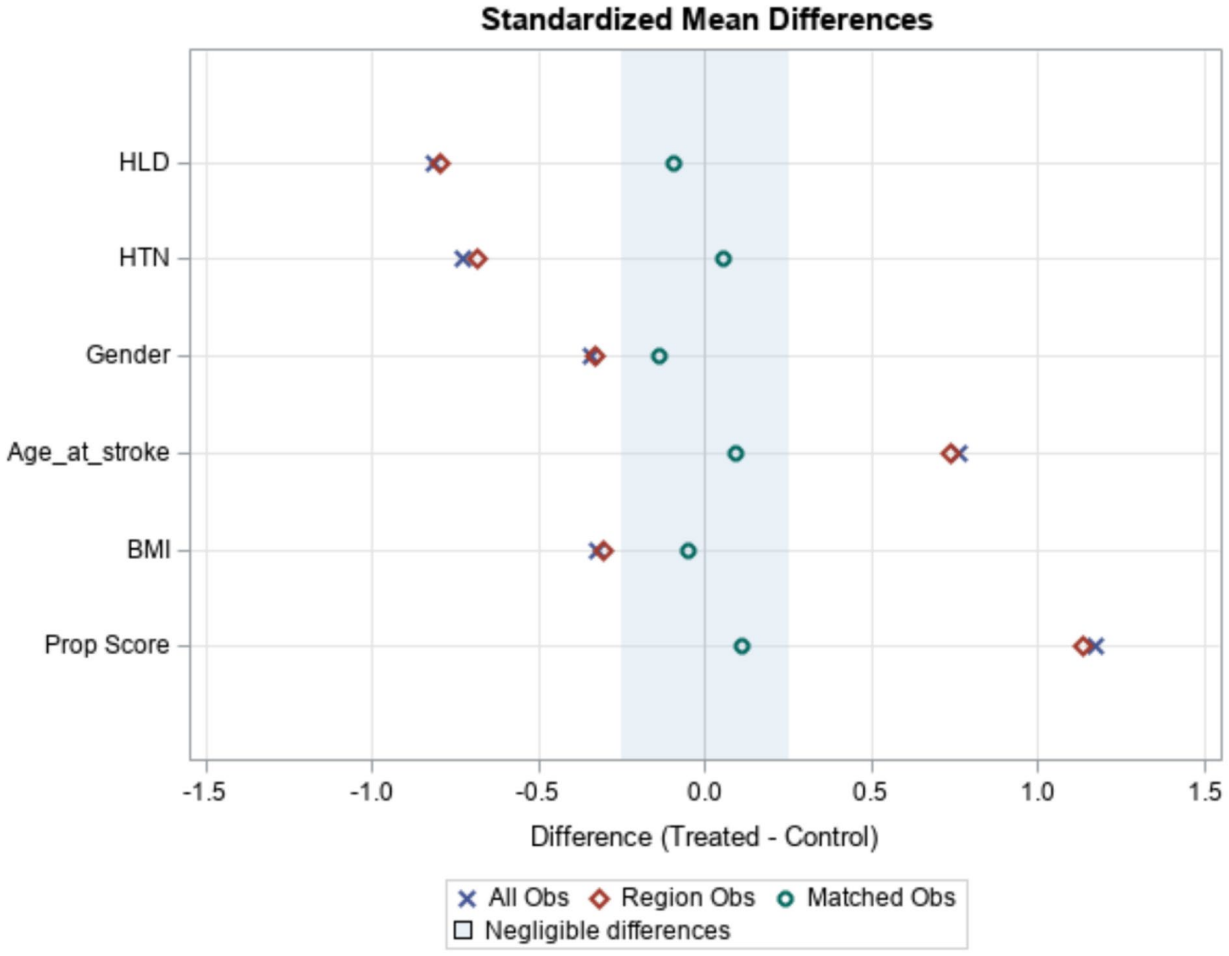
Matched patient demographic data.

Table 2 and Figure 1 present the demographic and clinical characteristics of the stroke and control patients after matching. There were 87 stroke and 77 control patients in the original data. The propensity score match was able to match 40 stroke patients to 40 control patients using a one-to-one match. There were no significant between-group differences in the matching characteristics or their Appalachian status. Figure 1 shows how, as a result of matching, the two groups became similar. The blue X’s and red diamonds represent differences prior to matching, and the green circles are differences after matching. The shaded region denotes differences that are negligible.

**Table 2:** Matched patient demographic data.

Demographics	Stroke	Control	*p*-value	Effect Size^1^
	*n* = 40	*n* = 40		
Age in years, mean (±SD)	63.8 (16.9)	61.9 (11.6)	0.555	0.131
BMI, mean (±SD)	29.3 (7.7)	30.5 (10.2)	0.569	0.133
Gender, *n* (%) Female	27 (67.5%)	27 (67.5%)	1	0
HTN, *n* (%)	33 (82.5%)	34 (85.0%)	0.762	0.034
HLD, *n* (%)	20 (50.0%)	18 (45.0%)	0.654	0.050
Appalachia, *n* (%)	27 (67.5%)	29 (72.5%)	0.626	0.055

1 Cohen’s d was used for continuous variables and φ (phi) for categorical.

### 3.2 Expression of VCID biomarkers among stroke and control patients

Expression of VCID biomarkers between stroke and control patients are shown in Table 3. GFAP (*p* < 0.001, Cliff’s d = 0.505) was increased in the stroke patients while AB 40 (*p* = 0.006, Cliff’s d = 0.363), A β 42 (*p* < 0.001, Cliff’s d = 0.450), and VEGFA (*p* = 0.005, Cliff’s d = 0.413) where increased in the control patients.

**Table 3:** Stroke vs. control comparison of VCID biomarkers.

Biomarker	Stroke	Control	*p*-value	Effect size^1^
	*n* = 40	*n* = 40		
A β 40	200.4 [153–256]	254 [214–319]	0.006	0.363
A β 42	13 [10.9–16]	17.6 [14.3–19.8]	< 0.001	0.450
tau	9.1 [6.1–12.4]	8.4 [5.8–11.1]	0.453	0.098
NfLight	11.8 [8.2–33.3]	14.3 [7.8–38]	0.824	0.030
GFAP	400 [204.4–1259.6]	166.6 [109.4–230.8]	< 0.001	0.505
pTau181	18.6 [12.3–25]	17.9 [14.2–21.5]	0.996	0.001
bFGF	13.3 [6.9–30.6]	17.4 [5.5–38.7]	0.596	0.072
FlT1	7.4 [2.1–13.5]	6.1 [2.1–11.3]	0.738	0.044
PlGF	5.7 [2.6–16.3]	7.9 [3.7–21.5]	0.214	0.165
VEGFA	16.6 [3.4–45.4]	46.6 [25.1–105.7]	0.005	0.413
VEGFC	24.3 [11.2–36.6]	24.4 [7.6–54.6]	0.763	0.040
VEGFD	7.3 [4.5–12.2]	8.5 [5.9–14.9]	0.192	0.172

Values represent median and interquartile range.

1 Cliff’s d.

### 3.3 Expression of VCID biomarkers among Appalachian and non-Appalachian patients

Table 4 displays ADRD biomarkers expression for Appalachian and non-Appalachian control patients. No biomarkers were significantly different between the control groups. The expression of four biomarkers were significantly different between stroke and control patients within the Appalachian population (Table 5). A β 40 (*p* = 0.005, Cliff’s d = 0.448), A β 42 (*p* < 0.001, Cliff’s d = 0.554), and VEGFA (*p* = 0.008, Cliff’s d = 0.459) were significantly elevated in control patients compared to stroke patients. GFAP (*p* = 0.010, Cliff’s d = 0.409) was significantly increased in stroke patients. Table 6 shows that within the non-Appalachian population only GFAP expression differed significantly between stroke and control patients. GFAP (*p* = 0.001, Cliff’s d = 0.818) was expressed significantly higher in stroke patients.

**Table 4:** Baseline expression of VCID biomarkers between Appalachian and Non-Appalachian control populations.

Biomarker	Appalachian control	Non-Appalachian control	*p*-value	Effect size^1^
	*n* = 29	*n* = 11		
A β 40	250 [214–320]	264 [224–318]	0.739	0.069
A β 42	18 [14–19]	18 [13–21]	0.820	0.047
Tau	8 [5–11]	9 [6–11]	0.467	0.150
NfLight	14 [8–31]	19 [11–40]	0.640	0.097
GFAP	154 [105–218]	225 [124–243]	0.224	0.253
pTau181	18 [14–19]	20 [14–22]	0.269	0.229
bFGF	17 [11–40]	14 [3–37]	0.525	0.138
FlT1	6 [2–11]	8 [2–11]	0.662	0.091
PlGF	7 [3–26]	8 [5–20]	0.760	0.064
VEGFA	40 [27–107]	53 [25–86]	1	0.056
VEGFC	20 [6–53]	34 [15–106]	0.798	0.193
VEGFD	8 [6–14]	10 [6–16]	0.371	0.110

Values represent median and interquartile range.

1 Cliff’s d.

**Table 5:** Appalachian stroke vs. control comparison of VCID biomarkers.

Biomarker	Appalachian stroke	Appalachian control	*p*-value	Effect size^1^
	*n* = 27	*n* = 29		
A β 40	182.4 [159.2–224]	250 [214–320]	0.005	0.448
A β 42	12.5 [9.9–14.1]	17.6 [14.3–18.6]	<0.001	0.554
tau	8.8 [6.2–12.2]	8.3 [5.5–10.5]	0.550	0.094
NfLight	11.8 [7.4–31.8]	14.2 [7.6–31.5]	0.883	0.023
GFAP	244.6 [142.2–1820.4]	153.8 [104.6–217.9]	0.010	0.409
pTau181	18.5 [12.3–25.2]	17.7 [14–19.2]	0.711	0.058
bFGF	12.7 [7.3–25.9]	17.4 [10.8–39.9]	0.332	0.160
FlT1	8.2 [3.5–14.7]	6.1 [2.2–11]	0.261	0.179
PlGF	5.1 [2.7–8.5]	7.5 [3.4–25.9]	0.248	0.185
VEGFA	11.1 [2.9–31.1]	40.3 [26.5–106.9]	0.008	0.459
VEGFC	21 [11.4–31.7]	20.5 [6.4–52.7]	0.901	0.020
VEGFD	7.7 [3.5–11.7]	8.1 [5.5–13.7]	0.350	0.148

Values represent median and interquartile range.

1 Cliff’s d.

**Table 6:** Non-Appalachian stroke vs. control comparisons of VCID biomarkers.

Biomarker	Non-Appalachian Stroke	Non-Appalachian Control	*p*-value	Effect size^1^
	*n* = 13	*n* = 11		
A β 40	240 [144.6–330]	264 [224–318]	0.543	0.147
A β 42	13.1 [11.4–19.4]	17.7 [13.1–21.2]	0.271	0.266
tau	10.3 [6.1–14.6]	9.1 [6.2–11.3]	0.750	0.077
NfLight	16.9 [8.4–39.1]	18.6 [11.4–39.8]	0.778	0.073
GFAP	878 [552–1259.6]	224.7 [124.2–242.8]	0.001	0.818
pTau181	18.7 [15.3–20.4]	20.2 [14.4–21.8]	0.543	0.147
bFGF	18.1 [2.7–42.8]	14 [3.2–37.4]	1	0.000
FlT1	2.7 [1.9–8.5]	8.2 [2.1–11.3]	0.259	0.273
PlGF	7.9 [1.7–25.1]	8.3 [5.3–19.9]	0.794	0.063
VEGFA	21.6 [14.1–103.8]	53.5 [25.1–86.1]	0.477	0.200
VEGFC	34.2 [10.1–41.1]	33.8 [15.2–105.9]	0.664	0.108
VEGFD	7.3 [4.9–12.2]	10 [6.3–16.3]	0.369	0.217

Values represent median and interquartile range.

1 Cliff’s d.

## 4 Discussion

Dementia continues to be one of the leading causes of disability after ischemic stroke and research aims to elucidate the molecular mechanisms connecting stroke to dementia. Identifying key biomarkers that are associated with dementia after stroke is essential for pinpointing patients at high risk for dementia and providing them with early and intensive cognitive rehabilitation therapy or pharmacological interventions. This study aimed to identify early-timepoint changes in arterial expression of proteins previously linked to Alzheimer’s dementia and VCID. Using CVD controls, we were able to compare the expression of several dementia biomarkers in stroke patients compared to controls matched via propensity scoring. Allowing for a clearer understanding of how the molecular response to stroke may lead to worsened cognitive outcomes.

A β 40, A β 42, GFAP, and VEGFA expression differed between stroke and control patients. Expression of A β 40, A β 42, and VEGFA were decreased in stroke patients compared to CVD controls, while GFAP was elevated in the stroke patients. The decrease in A β 40 and A β 42 was unexpected, especially when compared to previous literature that found Aβ elevated after acute stroke (Sriram et al., 2022; Lee et al., 2005). Aβ40 and A β 42, as stated in the cerebral amyloid hypothesis, accumulate to form plaques and cause neurodegeneration in AD. This accumulation could be an important connection between stroke and VCID. Sriram et al. proposed that higher accumulation of Aβ post-stroke could be because of impaired Aβ clearance due to dysregulation and increased permeability of the Blood Brain Barrier (BBB) after ischemic stroke (Sriram et al., 2022). Thus elevated Aβ has been associated with worsened cognitive impairment post-stroke (Chen et al., 2022; Chi et al., 2019).

Similarly to Aβ, VEGFA expression was lower in the stroke population compared to the control population. VEGF is a group of growth factors that are dysregulated after stroke. VEGF includes VEGF-A, B, C, and D. Typically, VEGF-A is involved in promoting angiogenesis and neurogenesis, acting as a neuroprotective agent post-ischemic stroke (Greenberg and Jin, 2013). The decrease in VEGFA within stroke patients is contrary to the existing literature which shows evidence of VEGFA increase after stroke (Slevin et al., 2000; Matsuo et al., 2013; Qi et al., 2007). The reduction rather than increase in A β 40, A β 42, and VEGFA within this cohort could be explained by the acute time-point at which the patient sample was obtained.

GFAP is a biomarker of neuroinflammation and an intermediate filament protein found on astrocyte cytoskeletons that is upregulated around Aβ plaques in AD patients (Kim et al., 2023). This study found GFAP elevated in stroke patients at an acute time point, consistent with previous literature. GFAP typically increases after stroke, reaching peak levels 48 h after stroke as a result of neural damage (Amalia, 2021). An increase in GFAP could indicate an increase in the presence of reactive astrocytes which overexpress GFAP (Kim et al., 2023). Reactive astrocytes have been linked to neuroinflammation in AD patients that precedes Aβ plaques, with clinical studies showing a significant increase in peripheral blood GFAP levels in dementia patients compared to healthy controls (Kim et al., 2023). Researchers have identified a positive correlation between increasing GFAP and functional deficits post ischemic stroke (Amalia, 2021).

The second aim of this study compared the expression of dementia biomarkers between stroke and control patients in Appalachian populations and those in non-Appalachian populations. In the Appalachian population stroke patients A β 40, A β 42, and VEGFA were decreased, while GFAP was elevated compared to CVD controls. In the groups of patients from non-Appalachian counties only GFAP was significantly altered between stroke and control patients, with its expression being elevated in the stroke patients. There were no significant baseline differences in the expression of A β 40, A β 42, or GFAP between the CVD controls of Appalachian and non-Apalachin patients. There was an elevation in VEGFA within the Appalachian population controls compared to non-Appalachian.

In a previous study, McLouth et al. found worsened functional outcomes based on NIHSS in Appalachian patients associated with several plasma proteins (ANG, LIF R, IL15RA, CXCL9, CXCL10, IL-13, IL-6, and THBS4) at the time of MT, despite no significant differences in risk factors for stroke between Appalachian and non-Appalachian stroke patients tested (McLouth et al., 2024). Many of the proteins identified in this study have been associated with poor outcomes and increased stroke severity. Given these findings the authors suggest that a contributing factor to the worsened cognitive outcomes in Appalachian patients could be delayed presentation for treatment within the Appalachian population (McLouth et al., 2024).

The differences found in biomarker expression among Appalachian and Non-Appalachian populations in this study could also be explained by the delayed time to treatment experienced by patients from Appalachia. The decreased levels of A β 40, A β 42, and VEGFA in the Appalachian stroke patients in this study are unexpected and not in alignment with the literature. As stated before, A β 40 and A β 42 elevations have been strongly associated with worsened cognitive outcomes and would be expected to elevate in the Appalachian stroke population. However, this discrepancy may be explained by the early time-point at which these proteins were measured.

The role of factors such as environmental, socioeconomic, and behavioral factors may influence differential protein expression between Appalachian and Non-Appalachian populations. The Appalachian region relied heavily on coal mining to support its economy, the process of mining pollutes both the water supply and air in the region (Zipper and Skousen, 2021). Appalachian counties also have increased food insecurity, poverty, and reduced access to healthcare (Appalachian Regional Commission, 2021). People in Appalachian have higher rates of obesity, higher tobacco usage (Horn et al., 2022), and lower rates of physical activity compared to national average (Hoogland et al., 2019). All these factors contribute to a population at increased risk for stroke (Dong et al., 2024).

Future directions of this study could explore the expression of other biomarkers connected to dementia. Current clinical practice guidelines set by Alzheimer’s Association recommend the use of blood based biomarkers (BBM) testing as part of diagnostic workup for AD in conjunction with comprehensive clinical evaluation, they are not yet used in a predictive capacity in the United States (Palmqvist et al., 2025). Metanalysis by Grande et al. underscores GFAP, ptau217, and NfL as emerging predictive biomarkers for dementia (Grande et al., 2025). Although this paper explored the expression of GFAP, NfL, and pTau181 in the acute time frame of ischemic stroke, ptau217 should be investigate in future studies. Literature suggests that pTau217 is a strong predictor of AD (Janelidze et al., 2023; Pandey et al., 2025; Xiao et al., 2024). Furthermore, Barthelemy et al. finds that pTau217 outperforms pTau181 as a biomarker for AD (Barthelemy et al., 2020). Another emerging field is the use of extracellular vesicles (EV) expression to predict dementia. Increased expression of EVs containing molecular biomarkers such as pTau and A β have been found to predict AD up to 10 years before clinical onset (Fiandaca et al., 2015). Recent metanalysis by Taha finds that EVs showed high diagnostic accuracy in differentiating AD and VCID (Taha, 2025). Given the diagnostic and predictive value of EVs, future studies are warranted to quantify the expression of EVs after stroke.

The study is limited by small sample size and absence of Intra and inter assay CV measurements. Future studies will also need to include more patients and controls, which will be made possible as the BACTRAC registry gathers more patient data. Currently, additional biomarker and cognitive data are being collected via BACTRAC. Additional timepoints to determine long-term changes in patient proteomic expression and cognitive consequences will also be explored.

## 5 Conclusion

This study demonstrates A β 40, A β 42, GFAP, and VEGFA are differentially expressed in stroke and CVD controls during mechanical thrombectomy. Furthermore, we present evidence demonstrating that there are proteomic differences between stroke and CVD controls based on the patients’ geographic region. The results of this study add to ongoing research to identify the expression of markers that can predict cognitive decline in stroke patients at an early time-point. Additionally, the investigation into the expression of ADRD and VCID biomarkers in Appalachian patients demonstrates that there may be molecular factors that influence stroke outcomes in this patient population.

## Data Availability

The original contributions presented in the study are included in the article/Supplementary material, further inquiries can be directed to the corresponding author.
